# 1,4-Bis(benz­yloxy)-2-*tert*-butyl­benzene

**DOI:** 10.1107/S1600536808003383

**Published:** 2008-02-22

**Authors:** Guo-wei Wang, Wen-yuan Wu, Ling-hua Zhuang, Jin-tang Wang

**Affiliations:** a Department of Applied Chemistry, College of Science, Nanjing University of Technology, Nanjing 210009, People’s Republic of China

## Abstract

The title compound, C_24_H_26_O_2_, was obtained unintentionally as the product of an attempted synthesis of a new chiral cobalt salen catalyst. There are no classical hydrogen bonds; inter­molecular C—H⋯π stacking inter­actions between aromatic rings help to establish the mol­ecular conformation.

## Related literature

For related literature, see: Annis & Jacobsen (1999 ▶); Kwon & Kim (2003 ▶); Ready & Jacobsen (2001 ▶). For bond-length data, see: Allen *et al.* (1987 ▶).
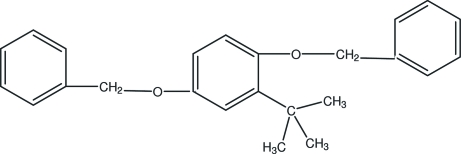


## Experimental

### 

#### Crystal data

C_24_H_26_O_2_
*M* *_r_* = 346.45
Monoclinic, 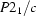

*a* = 6.5570 (13) Å
*b* = 23.772 (5) Å
*c* = 12.924 (3) Å
β = 93.21 (3)°
*V* = 2011.3 (7) Å^3^
*Z* = 4
Mo *K*α radiation
μ = 0.07 mm^−1^
*T* = 293 (2) K
0.40 × 0.30 × 0.20 mm

#### Data collection

Enraf–Nonius CAD-4 diffractometer
Absorption correction: ψ scan (North *et al.*, 1968 ▶) *T*_min_ = 0.969, *T*_max_ = 0.986
4285 measured reflections
3935 independent reflections
2336 reflections with *I* > 2σ(*I*)
*R*_int_ = 0.027
3 standard reflections every 200 reflections intensity decay: none

#### Refinement

*R*[*F* ^2^ > 2σ(*F* ^2^)] = 0.072
*wR*(*F* ^2^) = 0.162
*S* = 1.02
3935 reflections
188 parameters
H-atom parameters constrained
Δρ_max_ = 0.35 e Å^−3^
Δρ_min_ = −0.33 e Å^−3^

### 

Data collection: *CAD-4 Software* (Enraf–Nonius, 1989 ▶); cell refinement: *CAD-4 Software*; data reduction: *XCAD4* (Harms & Wocadlo, 1995 ▶); program(s) used to solve structure: *SHELXS97* (Sheldrick, 2008 ▶); program(s) used to refine structure: *SHELXL97* (Sheldrick, 2008 ▶); molecular graphics: *SHELXTL* (Sheldrick, 2008 ▶); software used to prepare material for publication: *SHELXTL*.

## Supplementary Material

Crystal structure: contains datablocks global, I. DOI: 10.1107/S1600536808003383/rk2077sup1.cif

Structure factors: contains datablocks I. DOI: 10.1107/S1600536808003383/rk2077Isup2.hkl

Additional supplementary materials:  crystallographic information; 3D view; checkCIF report

## Figures and Tables

**Table 1:** Hydrogen-bond geometry (Å, °)

*D*—H⋯*A*	*D*—H	H⋯*A*	*D*⋯*A*	*D*—H⋯*A*
C5—H5⋯*Cg*3^i^	0.93	2.96	3.725	141
C15—H15*C*⋯*Cg*3^ii^	0.96	2.87	3.818	168
C24—H24⋯*Cg*2^iii^	0.93	2.71	3.560	153

Symmetry codes: (i) 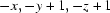; (ii) 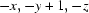; (iii) 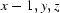. *Cg*2 is the centroid of atoms C8–C13 and *Cg*3 is the centroid of atoms C19–C24.

**Fig. 1.**
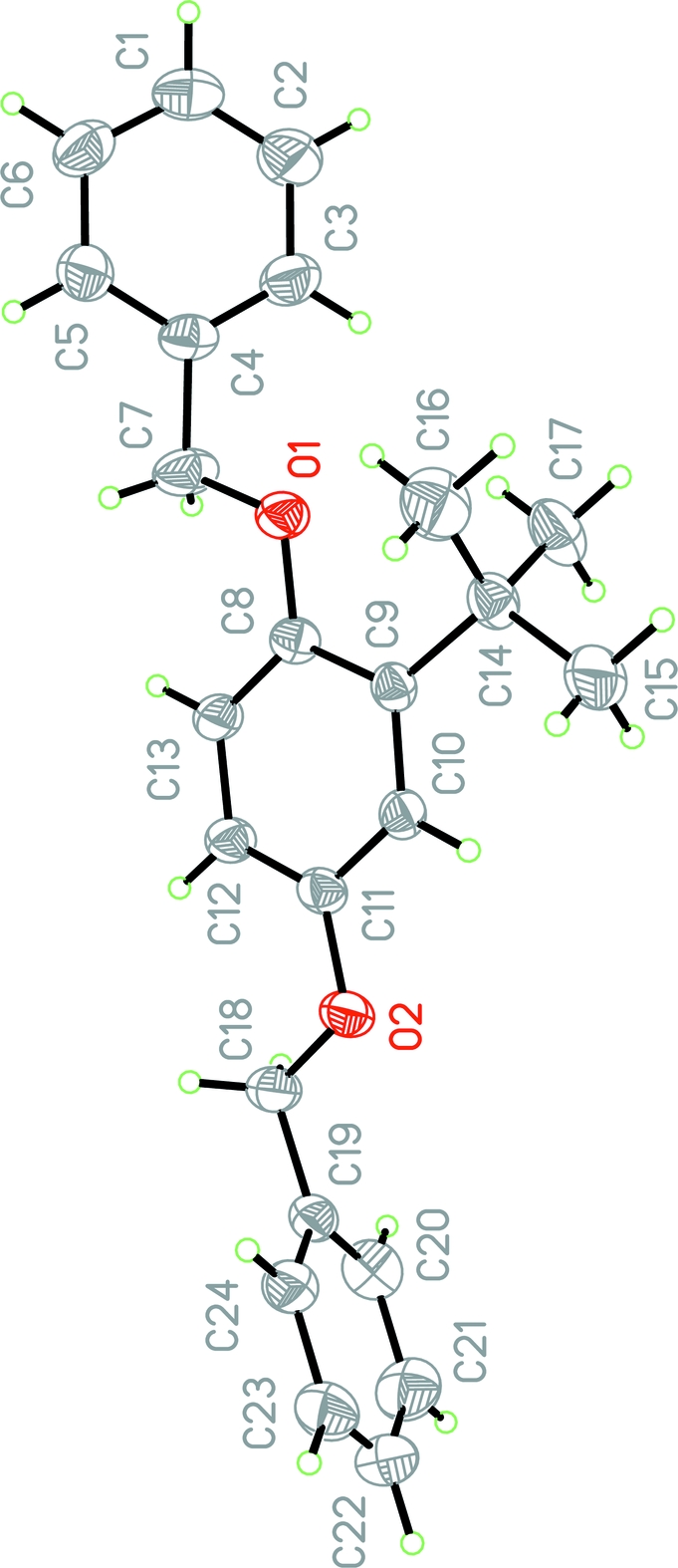
A view of the molecular structure of (I) with the atom numbering scheme. Displacement ellipsoids are drawn at 30% probability level. H atoms are presented as a small spheres of arbitrary radius.

**Fig. 2.**
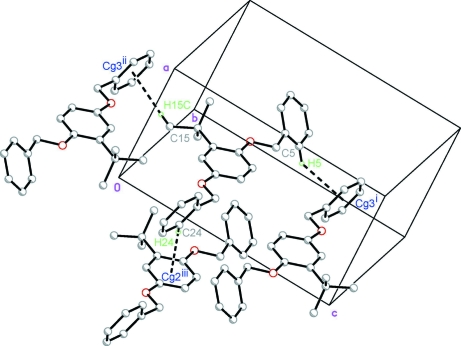
A packing diagram of (I) showing the intermolecular C—H···π interactions (dashed lines). The H atoms not involved in H–bonds are omitted for clarity. Cg2 and Cg3 denote the centroids of the C8–C13 and C19–C24 phenyl rings respectively. [Symmetry codes: (i) -x, -y + 1, -z + 1; (ii) -x, -y + 1, -z; (iii) x - 1, y, z.]

## supplementary crystallographic information

### Comment

Chiral Co(*salen*) complexes are widely used in the Hydrolytic Kinetic Resolution of terminal epoxides, such as epichlorohydrin (Annis & Jacobsen, 1999). Bis–phenols, such as 2–tert–butyl–hydroquinone, are useful as starting materials for the synthesis of salicylaldehyde (Ready & Jacobsen, 2001), especially in the synthesis of polymeric *salen* complexes (Kwon & Kim, 2003). We here report the molecular and crystal structure of the title compound (I).

The atom–numbering scheme of (I) is shown in Fig. 1. A l l bond lengths and angles are within normal ranges (Allen *et al.*, 1987). The molecule contains three phenyl rings - ring 1 (C1–C6), ring 2 (C8–C13), ring 3 (C19—C24), which are planar. The dihedral angle between ring 1 and ring 2 is 48.5 (4)°. There are no typical hydrogen bonds, while intermolecular C—H···π stacking interactions between aromatic rings help to stabilize the molecular conformation of compound (I).

### Experimental

Under N_2_ atmosphere, a mixture of 5.5 g (33 mmol) of 2–tert–butyl–hydroquinone, 2.25 g anhydrous potassium carbonate (16.0 mmol) in 75 ml dry acetonitrile were stirred for half an hour at room temperature. Subsequently 8.2 g (66 mmol) of benzyl chloride and 600 mg (3.6 mmol) of potassium iodide were added to the reaction mixture and was refluxed for 3 h in inert atmosphere. The mixture was cooled to room temperature and was filtrated and the solvent was removed under reduced pressure. The crude product was purified with *n*–hexane solution. Crystals of (I) suitable for *X*–ray diffraction were further recrystallized by slow evaporation of acetone. ^1^H NMR (CDCl_3_, δ, p.p.m.) 7.45 (q, 10H), 7.00 (s, 1H), 6.89 (d, 1H), 6.74 (d, 1H), 5.05 (s, 2H), 4.99 (S, 2H), 1.38 (s, 9H).

### Refinement

During the refinement, the phenyl rings were treated as rigid hexagons with C–C bond lengths of 1.39 Å. H atoms were positioned geometrically (C—H = 0.930, 0.970 and 0.960Å for aromatic, methylene and methyl H atoms respectively) and constrained to ride on their parent atoms, with *U*_iso_(H) = *xU*_eq_(C), where *x* = 1.5 for methyl and *x* = 1.2 for all other H atoms.

### Crystal data

**Table:**

C_24_H_26_O_2_	*F*_000_ = 744
*M**_r_* = 346.45	*D*_x_ = 1.144 Mg m^−^^3^
Monoclinic, *P*2_1_/*c*	Mo *K*α radiation λ = 0.71073 Å
Hall symbol: -P 2ybc	Cell parameters from 25 reflections
*a* = 6.5570 (13) Å	θ = 9–13º
*b* = 23.772 (5) Å	µ = 0.07 mm^−^^1^
*c* = 12.924 (3) Å	*T* = 293 (2) K
β = 93.21 (3)º	Block, colourless
*V* = 2011.3 (7) Å^3^	0.40 × 0.30 × 0.20 mm
*Z* = 4	

### Data collection

**Table:**

Enraf–Nonius CAD-4 diffractometer	*R*_int_ = 0.027
Radiation source: fine–focus sealed tube	θ_max_ = 26.0º
Monochromator: graphite	θ_min_ = 1.7º
*T* = 293(2) K	*h* = 0→8
ω/2θ scans	*k* = 0→29
Absorption correction: ψ scan(North *et al.*, 1968)	*l* = −15→15
*T*_min_ = 0.969, *T*_max_ = 0.986	3 standard reflections
4285 measured reflections	every 200 reflections
3935 independent reflections	intensity decay: none
2336 reflections with *I* > 2σ(*I*)	

### Refinement

**Table:**

Refinement on *F*^2^	Hydrogen site location: inferred from neighbouring sites
Least-squares matrix: full	H-atom parameters constrained
*R*[*F*^2^ > 2σ(*F*^2^)] = 0.072	*w* = 1/[σ^2^(*F*_o_^2^) + (0.0054*P*)^2^ + 4.1075*P*] where *P* = (*F*_o_^2^ + 2*F*_c_^2^)/3
*wR*(*F*^2^) = 0.162	(Δ/σ)_max_ < 0.001
*S* = 1.03	Δρ_max_ = 0.35 e Å^−^^3^
3935 reflections	Δρ_min_ = −0.33 e Å^−^^3^
188 parameters	Extinction correction: SHELXL97 (Sheldrick, 2008), Fc^*^=kFc[1+0.001xFc^2^λ^3^/sin(2θ)]^-1/4^
Primary atom site location: structure-invariant direct methods	Extinction coefficient: 0.0384 (15)
Secondary atom site location: difference Fourier map	

### Special details

**Table:**

Geometry. All s.u.'s (except the s.u.'s in the dihedral angle between two l.s. planes) are estimated using the full covariance matrix. The cell s.u.'s are taken into account individually in the estimation of s.u.'s in distances, angles and torsion angles; correlations between s.u.'s in cell parameters are only used when they are defined by crystal symmetry. An approximate (isotropic) treatment of cell s.u.'s is used for estimating s.u.'s involving l.s. planes.
Refinement. Refinement of *F*^2^ against ALL reflections. The weighted *R*–factor *wR* and goodness of fit *S* are based on *F*^2^, conventional *R*–factors *R* are based on *F*, with *F* set to zero for negative *F*^2^. The threshold expression of *F*^2^ > 2σ(*F*^2^) is used only for calculating *R*–factors(gt) *etc*. and is not relevant to the choice of reflections for refinement. *R*–factors based on *F*^2^ are statistically about twice as large as those based on *F*, and *R*–factors based on ALL data will be even larger.

### Fractional atomic coordinates and isotropic or equivalent isotropic displacement parameters (Å^2^)

**Table:**

	*x*	*y*	*z*	*U*_iso_*/*U*_eq_	
C1	1.0565 (4)	0.27733 (12)	0.5023 (3)	0.0843 (15)	
H1	1.1645	0.2523	0.5133	0.101*	
C2	1.0574 (4)	0.31573 (15)	0.4212 (3)	0.0931 (17)	
H2	1.1661	0.3164	0.3780	0.112*	
C3	0.8960 (4)	0.35314 (12)	0.4048 (2)	0.0765 (13)	
H3	0.8966	0.3788	0.3505	0.092*	
C4	0.7335 (4)	0.35215 (11)	0.4694 (2)	0.0582 (11)	
C5	0.7326 (4)	0.31375 (13)	0.55046 (19)	0.0719 (13)	
H5	0.6239	0.3131	0.5937	0.086*	
C6	0.8941 (5)	0.27634 (11)	0.5669 (2)	0.0777 (14)	
H6	0.8934	0.2506	0.6212	0.093*	
C7	0.5631 (7)	0.3943 (2)	0.4568 (3)	0.0746 (14)	
H7A	0.4514	0.3831	0.4984	0.090*	
H7B	0.6112	0.4309	0.4806	0.090*	
C8	0.3396 (3)	0.43592 (8)	0.32489 (12)	0.0461 (9)	
C9	0.2627 (2)	0.43853 (6)	0.22256 (11)	0.0447 (8)	
C10	0.1057 (2)	0.47575 (8)	0.19454 (11)	0.0475 (9)	
H10	0.0543	0.4775	0.1261	0.057*	
C11	0.0255 (3)	0.51035 (8)	0.26886 (14)	0.0486 (9)	
C12	0.1024 (3)	0.50774 (8)	0.37119 (13)	0.0530 (10)	
H12	0.0487	0.5309	0.4209	0.064*	
C13	0.2594 (3)	0.47053 (9)	0.39920 (10)	0.0547 (10)	
H13	0.3108	0.4688	0.4677	0.066*	
C14	0.3591 (3)	0.40301 (9)	0.13571 (14)	0.0655 (10)	
C15	0.2469 (4)	0.41208 (13)	0.02966 (13)	0.0934 (18)	
H15A	0.2507	0.4512	0.0118	0.140*	
H15B	0.1074	0.4002	0.0326	0.140*	
H15C	0.3123	0.3905	−0.0218	0.140*	
C16	0.3501 (8)	0.34034 (19)	0.1595 (4)	0.0885 (16)	
H16A	0.4201	0.3331	0.2254	0.133*	
H16B	0.4145	0.3197	0.1065	0.133*	
H16C	0.2101	0.3288	0.1616	0.133*	
C17	0.5827 (6)	0.4212 (2)	0.1272 (4)	0.0834 (15)	
H17A	0.6565	0.4156	0.1927	0.125*	
H17B	0.5875	0.4602	0.1085	0.125*	
H17C	0.6439	0.3990	0.0751	0.125*	
C18	−0.2037 (6)	0.58533 (19)	0.3026 (3)	0.0689 (13)	
H18A	−0.0948	0.6094	0.3307	0.083*	
H18B	−0.2610	0.5653	0.3596	0.083*	
C19	−0.3651 (2)	0.61998 (7)	0.24720 (11)	0.0528 (10)	
C20	−0.3295 (3)	0.67607 (6)	0.2238 (2)	0.0705 (12)	
H20	−0.2036	0.6923	0.2422	0.085*	
C21	−0.4821 (3)	0.70793 (7)	0.1730 (2)	0.0846 (15)	
H21	−0.4583	0.7455	0.1574	0.102*	
C22	−0.6703 (3)	0.68368 (9)	0.14560 (14)	0.0824 (15)	
H22	−0.7724	0.7050	0.1116	0.099*	
C23	−0.7060 (2)	0.62759 (9)	0.1690 (2)	0.0797 (14)	
H23	−0.8319	0.6114	0.1506	0.096*	
C24	−0.5534 (2)	0.59573 (8)	0.21979 (18)	0.0636 (11)	
H24	−0.5772	0.5582	0.2354	0.076*	
O1	0.4937 (3)	0.39766 (9)	0.35168 (17)	0.0581 (7)	
O2	−0.1255 (2)	0.54656 (6)	0.23203 (9)	0.0577 (7)	

### Atomic displacement parameters (Å^2^)

**Table:**

	*U*^11^	*U*^22^	*U*^33^	*U*^12^	*U*^13^	*U*^23^
C1	0.061 (3)	0.083 (4)	0.107 (4)	0.019 (3)	−0.014 (3)	0.000 (3)
C2	0.056 (3)	0.102 (4)	0.123 (5)	0.014 (3)	0.013 (3)	0.024 (4)
C3	0.061 (3)	0.078 (3)	0.089 (3)	0.009 (2)	0.002 (2)	0.021 (3)
C4	0.058 (2)	0.070 (2)	0.055 (2)	0.013 (2)	−0.0180 (19)	−0.015 (2)
C5	0.076 (3)	0.084 (3)	0.056 (3)	0.013 (3)	−0.001 (2)	−0.003 (2)
C6	0.096 (4)	0.070 (3)	0.065 (3)	0.014 (3)	−0.014 (3)	0.008 (2)
C7	0.081 (3)	0.082 (3)	0.058 (3)	0.032 (3)	−0.023 (2)	−0.013 (2)
C8	0.0405 (19)	0.051 (2)	0.046 (2)	−0.0003 (17)	−0.0038 (15)	−0.0029 (17)
C9	0.0414 (19)	0.049 (2)	0.0437 (19)	−0.0036 (17)	0.0049 (15)	−0.0008 (16)
C10	0.0413 (19)	0.061 (2)	0.0397 (19)	−0.0004 (18)	−0.0004 (15)	−0.0006 (17)
C11	0.0343 (18)	0.058 (2)	0.053 (2)	−0.0001 (17)	0.0006 (16)	0.0002 (18)
C12	0.057 (2)	0.057 (2)	0.044 (2)	0.0122 (19)	0.0000 (17)	−0.0064 (18)
C13	0.055 (2)	0.066 (3)	0.041 (2)	0.015 (2)	−0.0061 (17)	−0.0015 (18)
C14	0.065 (2)	0.075 (3)	0.057 (2)	0.002 (2)	0.0035 (17)	−0.0086 (19)
C15	0.100 (4)	0.131 (5)	0.049 (3)	0.039 (3)	−0.004 (2)	−0.020 (3)
C16	0.114 (4)	0.071 (3)	0.081 (3)	0.001 (3)	0.009 (3)	−0.021 (3)
C17	0.065 (3)	0.112 (4)	0.076 (3)	0.001 (3)	0.028 (2)	−0.009 (3)
C18	0.066 (3)	0.090 (3)	0.051 (2)	0.032 (2)	−0.001 (2)	−0.004 (2)
C19	0.051 (2)	0.063 (3)	0.045 (2)	0.0094 (19)	0.0053 (17)	−0.0049 (18)
C20	0.069 (3)	0.066 (3)	0.076 (3)	−0.012 (2)	0.000 (2)	−0.012 (2)
C21	0.109 (4)	0.054 (3)	0.091 (4)	0.011 (3)	0.005 (3)	0.008 (3)
C22	0.077 (3)	0.096 (4)	0.073 (3)	0.032 (3)	0.000 (3)	0.010 (3)
C23	0.050 (3)	0.107 (4)	0.082 (3)	0.002 (3)	0.003 (2)	0.004 (3)
C24	0.054 (2)	0.066 (3)	0.072 (3)	0.002 (2)	0.008 (2)	0.007 (2)
O1	0.0566 (16)	0.0708 (18)	0.0461 (14)	0.0190 (14)	−0.0045 (12)	−0.0035 (13)
O2	0.0476 (15)	0.0733 (18)	0.0516 (15)	0.0168 (13)	−0.0015 (12)	−0.0063 (13)

### Geometric parameters (Å, °)

**Table:**

C1—C2	1.3900	C14—C16	1.523 (5)
C1—C6	1.3900	C14—C15	1.5341
C1—H1	0.9300	C14—C17	1.539 (5)
C2—C3	1.3900	C15—H15A	0.9600
C2—H2	0.9300	C15—H15B	0.9600
C3—C4	1.3900	C15—H15C	0.9600
C3—H3	0.9300	C16—H16A	0.9600
C4—C5	1.3900	C16—H16B	0.9600
C4—C7	1.503 (4)	C16—H16C	0.9600
C5—C6	1.3900	C17—H17A	0.9600
C5—H5	0.9300	C17—H17B	0.9600
C6—H6	0.9300	C17—H17C	0.9600
C7—O1	1.411 (4)	C18—O2	1.413 (4)
C7—H7A	0.9700	C18—C19	1.492 (4)
C7—H7B	0.9700	C18—H18A	0.9700
C8—O1	1.3888	C18—H18B	0.9700
C8—C9	1.3900	C19—C20	1.3900
C8—C13	1.3900	C19—C24	1.3900
C9—C10	1.3900	C20—C21	1.3900
C9—C14	1.5657	C20—H20	0.9300
C10—C11	1.3900	C21—C22	1.3900
C10—H10	0.9300	C21—H21	0.9300
C11—O2	1.3766	C22—C23	1.3900
C11—C12	1.3900	C22—H22	0.9300
C12—C13	1.3900	C23—C24	1.3900
C12—H12	0.9300	C23—H23	0.9300
C13—H13	0.9300	C24—H24	0.9300
			
C2—C1—C6	120.0	C15—C14—C9	111.8
C2—C1—H1	120.0	C17—C14—C9	108.9 (2)
C6—C1—H1	120.0	C14—C15—H15A	109.5
C1—C2—C3	120.0	C14—C15—H15B	109.5
C1—C2—H2	120.0	H15A—C15—H15B	109.5
C3—C2—H2	120.0	C14—C15—H15C	109.5
C4—C3—C2	120.0	H15A—C15—H15C	109.5
C4—C3—H3	120.0	H15B—C15—H15C	109.5
C2—C3—H3	120.0	C14—C16—H16A	109.5
C5—C4—C3	120.0	C14—C16—H16B	109.5
C5—C4—C7	119.0 (3)	H16A—C16—H16B	109.5
C3—C4—C7	120.9 (3)	C14—C16—H16C	109.5
C4—C5—C6	120.0	H16A—C16—H16C	109.5
C4—C5—H5	120.0	H16B—C16—H16C	109.5
C6—C5—H5	120.0	C14—C17—H17A	109.5
C5—C6—C1	120.0	C14—C17—H17B	109.5
C5—C6—H6	120.0	H17A—C17—H17B	109.5
C1—C6—H6	120.0	C14—C17—H17C	109.5
O1—C7—C4	109.9 (3)	H17A—C17—H17C	109.5
O1—C7—H7A	109.7	H17B—C17—H17C	109.5
C4—C7—H7A	109.7	O2—C18—C19	108.9 (3)
O1—C7—H7B	109.7	O2—C18—H18A	109.9
C4—C7—H7B	109.7	C19—C18—H18A	109.9
H7A—C7—H7B	108.2	O2—C18—H18B	109.9
O1—C8—C9	119.10	C19—C18—H18B	109.9
O1—C8—C13	120.88	H18A—C18—H18B	108.3
C9—C8—C13	120.0	C20—C19—C24	120.0
C10—C9—C8	120.0	C20—C19—C18	120.7 (2)
C10—C9—C14	118.6	C24—C19—C18	119.3 (2)
C8—C9—C14	121.2	C21—C20—C19	120.0
C11—C10—C9	120.0	C21—C20—H20	120.0
C11—C10—H10	120.0	C19—C20—H20	120.0
C9—C10—H10	120.0	C20—C21—C22	120.0
O2—C11—C10	115.07	C20—C21—H21	120.0
O2—C11—C12	124.91	C22—C21—H21	120.0
C10—C11—C12	120.0	C21—C22—C23	120.0
C13—C12—C11	120.0	C21—C22—H22	120.0
C13—C12—H12	120.0	C23—C22—H22	120.0
C11—C12—H12	120.0	C24—C23—C22	120.0
C12—C13—C8	120.0	C24—C23—H23	120.0
C12—C13—H13	120.0	C22—C23—H23	120.0
C8—C13—H13	120.0	C23—C24—C19	120.0
C16—C14—C15	107.2 (2)	C23—C24—H24	120.0
C16—C14—C17	109.7 (3)	C19—C24—H24	120.0
C15—C14—C17	108.0 (2)	C8—O1—C7	117.9 (2)
C16—C14—C9	111.2 (2)	C11—O2—C18	117.68 (19)
			
C6—C1—C2—C3	0.0	C10—C9—C14—C16	−124.7 (3)
C1—C2—C3—C4	0.0	C8—C9—C14—C16	59.3 (3)
C2—C3—C4—C5	0.0	C10—C9—C14—C15	−4.9
C2—C3—C4—C7	176.5 (3)	C8—C9—C14—C15	179.1
C3—C4—C5—C6	0.0	C10—C9—C14—C17	114.4 (2)
C7—C4—C5—C6	−176.6 (3)	C8—C9—C14—C17	−61.6 (2)
C4—C5—C6—C1	0.0	O2—C18—C19—C20	−107.8 (3)
C2—C1—C6—C5	0.0	O2—C18—C19—C24	72.8 (3)
C5—C4—C7—O1	−135.2 (3)	C24—C19—C20—C21	0.0
C3—C4—C7—O1	48.2 (4)	C18—C19—C20—C21	−179.40 (18)
O1—C8—C9—C10	178.7	C19—C20—C21—C22	0.0
C13—C8—C9—C10	0.0	C20—C21—C22—C23	0.0
O1—C8—C9—C14	−5.3	C21—C22—C23—C24	0.0
C13—C8—C9—C14	176.0	C22—C23—C24—C19	0.0
C8—C9—C10—C11	0.0	C20—C19—C24—C23	0.0
C14—C9—C10—C11	−176.1	C18—C19—C24—C23	179.41 (18)
C9—C10—C11—O2	178.3	C9—C8—O1—C7	−177.5 (3)
C9—C10—C11—C12	0.0	C13—C8—O1—C7	1.2 (4)
O2—C11—C12—C13	−178.2	C4—C7—O1—C8	−178.2 (3)
C10—C11—C12—C13	0.0	C10—C11—O2—C18	−176.0 (2)
C11—C12—C13—C8	0.0	C12—C11—O2—C18	2.3 (3)
O1—C8—C13—C12	−178.7	C19—C18—O2—C11	−179.7 (2)
C9—C8—C13—C12	0.0		

### Hydrogen-bond geometry (Å, °)

**Table:**

*D*—H···*A*	*D*—H	H···*A*	*D*···*A*	*D*—H···*A*
C5—H5···Cg3^i^	0.93	2.96	3.725	141
C15—H15C···Cg3^ii^	0.96	2.87	3.818	168
C24—H24···Cg2^iii^	0.93	2.71	3.560	153

Symmetry codes: (i) −*x*, −*y*+1, −*z*+1; (ii) −*x*, −*y*+1, −*z*; (iii) *x*−1, *y*, *z*.
